# The optimal design of stepped wedge trials with equal allocation to sequences and a comparison to other trial designs

**DOI:** 10.1177/1740774517723921

**Published:** 2017-08-10

**Authors:** Jennifer A Thompson, Katherine Fielding, James Hargreaves, Andrew Copas

**Affiliations:** 1 Department of Infectious Disease Epidemiology, London School of Hygiene & Tropical Medicine, London, UK; 2 London Hub for Trials Methodology Research, MRC Clinical Trials Unit at University College London, London, UK; 3 Department of Social and Environmental Health Research, London School of Hygiene & Tropical Medicine, London, UK

**Keywords:** Stepped wedge trial, cluster randomised trial, hybrid trial, sample size, design effect, power, study design

## Abstract

**Background/Aims:**

We sought to optimise the design of stepped wedge trials with an equal allocation of clusters to sequences and explored sample size comparisons with alternative trial designs.

**Methods:**

We developed a new expression for the design effect for a stepped wedge trial, assuming that observations are equally correlated within clusters and an equal number of observations in each period between sequences switching to the intervention. We minimised the design effect with respect to (1) the fraction of observations before the first and after the final sequence switches (the periods with all clusters in the control or intervention condition, respectively) and (2) the number of sequences. We compared the design effect of this optimised stepped wedge trial to the design effects of a parallel cluster-randomised trial, a cluster-randomised trial with baseline observations, and a hybrid trial design (a mixture of cluster-randomised trial and stepped wedge trial) with the same total cluster size for all designs.

**Results:**

We found that a stepped wedge trial with an equal allocation to sequences is optimised by obtaining all observations after the first sequence switches and before the final sequence switches to the intervention; this means that the first sequence remains in the control condition and the last sequence remains in the intervention condition for the duration of the trial. With this design, the optimal number of sequences is $1/(1-\sqrt{R})$, where R=ρm/(1+ρ(m−1)) is the cluster-mean correlation, ρ is the intracluster correlation coefficient, and *m* is the total cluster size. The optimal number of sequences is small when the intracluster correlation coefficient and cluster size are small and large when the intracluster correlation coefficient or cluster size is large. A cluster-randomised trial remains more efficient than the optimised stepped wedge trial when the intracluster correlation coefficient or cluster size is small. A cluster-randomised trial with baseline observations always requires a larger sample size than the optimised stepped wedge trial. The hybrid design can always give an equally or more efficient design, but will be at most 5% more efficient. We provide a strategy for selecting a design if the optimal number of sequences is unfeasible. For a non-optimal number of sequences, the sample size may be reduced by allowing a proportion of observations before the first or after the final sequence has switched.

**Conclusion:**

The standard stepped wedge trial is inefficient. To reduce sample sizes when a hybrid design is unfeasible, stepped wedge trial designs should have no observations before the first sequence switches or after the final sequence switches.

## Introduction

Stepped wedge trials (SWTs) are growing in popularity, but modification of the design to minimise their sample size have not been fully explored.

**Figure 1.**
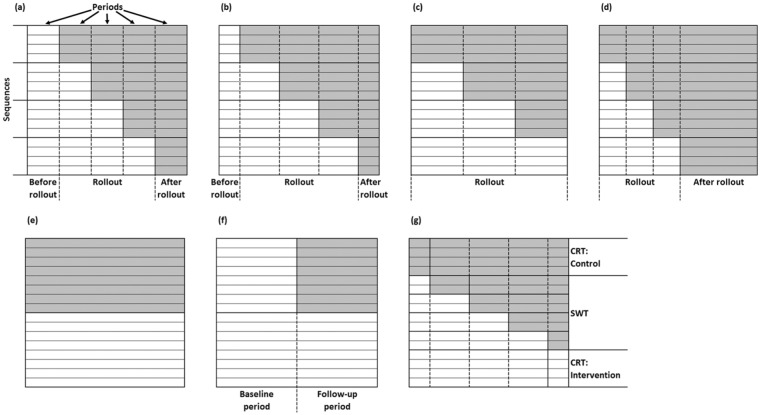
Diagrammatic illustrations of trial designs. Each has the same number of clusters and the same total cluster size. (a)–(d) Stepped wedge cluster-randomised trials (SWTs) with four sequences varying the amount of data before and after rollout. (a) Standard design: the same number of observations before and after rollout and between sequences switching, (b) number of observations before and after rollout is half the number between sequences switching, (c) optimised design: no observations before or after rollout, (d) no observations before rollout, 50% after rollout. (e)–(g) Other designs: (e) parallel cluster-randomised trial: CRT, (f) parallel cluster-randomised trial with baseline observations, (g) hybrid design with 50% CRT, 50% SWT with four sequences and the number of observations before and after rollout equal to half the number between sequences switching.

In an SWT, clusters are randomised into allocation sequences. Sequences consist of a different number of periods in the control condition, followed by the remaining periods of the trial in the intervention condition. At the beginning of each period, one of the sequences switches to the intervention, as shown in Figure 1(a). This means that a design with *k* sequences has *k* − 1 periods between the first sequence switching and the final sequence switching to the intervention condition. We will call this section of the trial ‘rollout’ because the intervention has been introduced to some but not all the clusters.

Before and after rollout, that is, before the first sequence switches to the intervention and after the final sequence switches to the intervention, there can be additional periods of data collection that may be longer or shorter than the periods during rollout (Figure 1(b) and (d)). In a standard SWT design (Figure 1(a)), these periods are the same length as the periods between rollout. Variations of the SWT design could include only the rollout period (Figure 1(c)), a period before rollout but not after or after rollout but not before.

There are many further possible variations but in this article, we only consider designs where the same number of observations is collected in each of the periods during rollout and that the same number of clusters is randomised to each sequence. This implies that the rollout will occur at an even pace, that is, equal numbers of clusters implement the intervention at each time, which we feel is a natural constraint when there are limited resources to conduct the implementation. We focus on data with equal correlation within each cluster.^1^

There are several approaches to sample size calculation for SWTs;^2–5^ the simplest is the design effect approach. Here, a sample size is calculated assuming individual randomisation and is then multiplied by a design effect to increase the sample size appropriately for a different design. Woertman et al. developed what they termed a design effect for an SWT, but this must be multiplied by the number of periods in the trial to give what we define here as the design effect.^2,6^ While their design effect has been a useful contribution to the literature, it is difficult to untangle the effects of each design component on the sample size to examine how to improve the efficiency of SWTs.

One such component which cannot be examined by the design effect of Woertman et al. is the number of periods before and after rollout; changing the number of periods before rollout increases the total cluster size so it is difficult to examine the impact of this change holding the total cluster size constant.^2^ Girling and Hemming found that having half a period before rollout and half a period after rollout produced greater efficiency than the standard design when the total cluster size was held constant,^7^ but it is not known whether even fewer observations before and after rollout would be more efficient. In addition, although there is a consensus through empirical evidence that the power of a standard SWT increases with an increase in the number of sequences,^2,3^ this has not been explored for variations of the SWT design.

Researchers often cite increased statistical power as a reason for choosing SWTs over other trial designs.^8^ Designs where clusters act as their own control can be more powerful,^7^ but they also require assumptions about changes in the outcome over time. Comparisons have been made between SWTs and parallel cluster-randomised trials (CRTs; Figure 1(e)), CRTs with baseline observations where half of the total cluster size are baseline observations (Figure 1(f)), and more recently with the hybrid design described by Girling and Hemming.^7^ The hybrid design includes sequences that are in the control or intervention conditions for the entire study and allows allocation to those two sequences to differ from allocation to the remaining sequences which form an SWT design, as shown in Figure 1(g). Standard SWT designs have been found to be more efficient than CRTs when the intracluster correlation coefficient (ICC) is high and when the total cluster sizes are large, and a standard SWT with four or more sequences always has more power than a CRT with baseline observations.^6,9–11^ The hybrid design appears to have the highest power as it is most flexible,^7^ but the degree of efficiency gain from allowing unequal allocation has not been established.

In this article, we give a new design effect expression for an SWT that allows the number of observations before and after rollout to vary without increasing the total cluster size but maintains the requirement common to the standard SWT of equal-sized periods between sequences switching to the intervention and the same number of clusters randomised to each sequence. This allowed us to identify the optimal number of sequences and the optimal number of observations before and after rollout to minimise the required number of clusters for a given power, ICC, and total cluster size. We compare the efficiency of our optimised SWT designs to several other common trial designs for a given power, ICC, and total cluster size, and we provide guidance in choosing a trial design. An example is then used to demonstrate the difference in sample size between possible designs.

## Methods

### SWT

Woertman et al. developed a design effect for an SWT under the assumptions of the Hussey and Hughes analysis model.^2,3,6^ We rewrite this design effect based on similar methodology to that used by Woertman et al.^2^ In our new design effect, the number of observations before and after rollout is specified as proportions of the total cluster size. For example, one could have half of all observations after rollout and none before rollout, as shown in Figure 1(d).

For simplicity, we assumed that the outcome is normally distributed, clusters are of equal size, and observations are equally correlated within clusters regardless of time or whether from the control or intervention condition. We assume that the intervention effect is constant over time, is fully realised by the first observation after the intervention is implemented, and is common across all clusters. We also require that secular trends are common to all clusters, the same number of clusters is randomised to each sequence, and that there is the same number of observations in all periods between sequences switching to the intervention.

This new design effect will be used to find the combination of number of sequences and proportion of observations before and after rollout that minimise the sample size (number of clusters) for a given power, total cluster size, and ICC. This SWT, derived under the constraint of equal allocation to sequences, will be referred to as an ‘optimised’ SWT.

This optimised SWT will then be compared to other trial designs. We will consider a CRT, a CRT with baseline observations and the hybrid design.^7^ Throughout these comparisons, we fix the power, total cluster size, and ICC.

### Parallel CRT

A CRT (Figure 1(e)) is an attractive design because the intervention effect is not confounded with time and so it does not require assumptions about secular trends. The published design effect for a CRT is as follows

(1) $$DE_{\mathrm{CRT}}=1+\left(m-1\right)\rho$$

where *m* is the total cluster size, and ρ is the ICC.^12^

### Parallel CRT with baseline observations

A CRT with baseline observations (Figure 1(f)) is equivalent to an SWT with two sequences, some proportion of observations before rollout and no observations after rollout.^13^ Making the same assumptions as the SWT, such a design can be analysed with the same model as an SWT,^3^ and so, the new design effect can also be applied. We used our design effect to find the optimum proportion of observations to have at baseline to minimise the sample size of this design before comparing the required sample size to the optimised SWT.

### Hybrid design trial

Girling and Hemming described a trial design where some of the clusters were randomised to a parallel CRT, while the remaining clusters were randomised to an SWT with half a period before rollout and half a period after rollout (Figure 1(g)).^7^ This hybrid trial design makes the same assumptions as the SWT and can be analysed with the Hussey and Hughes analysis model.^3^ They found that the optimal proportion of clusters to randomise to the SWT was the cluster-mean correlation defined as follows^7^

$$R=\frac{m\rho }{1+\left(m-1\right)\rho }$$

where 0≤R≤1 increases as the ICC or total cluster size increases. So, when the ICC or cluster size increases, the optimal proportion of clusters randomised to the SWT increases and the proportion randomised to the CRT reduces.

The hybrid design is flexible enough that it can simplify to a parallel CRT, it can simplify to a design similar to a standard SWT but with half a period before and half a period after rollout (Figure 1(b)), and it can simplify to a modified SWT design with no period before and after rollout, and the first and final periods are half the size of the other periods, similar to the design considered later in this article. The first two of these simplifications are straightforward to see; all clusters are randomised to the relevant part of the trial. The final simplification requires a proportion of 2/(*k* + 2) clusters to be randomised to the parallel CRT and the remaining clusters to be randomised to the SWT with *k* sequences. Following the recommendations of Girling and Hemming will lead to one of these designs if it is the most efficient option or it will lead to a hybrid design if that is most efficient.^7^

We compared our optimised SWT with *k* sequences to an optimal hybrid design to see whether the increased flexibility of the hybrid design gave a practically relevant decrease in sample size. The optimal hybrid had a proportion equal to the cluster-mean correlation of clusters randomised to the SWT, and the SWT within the hybrid had as many sequences as there were clusters.

### Choosing an SWT design

Finally, we acknowledge that the optimised SWT may not always be a practical design and provide recommendations for how to design an efficient and practical trial. We provide an example to demonstrate the differences in sample size of different designs.

## Results

### The design effect for an SWT

We define *k* as the number of sequences, β as the proportion of the total cluster size that is before rollout, and α the proportion of the total cluster size that is after rollout. For example, in a standard SWT, β=α=1/(k+1) (Figure 1(a)), alternatively one could have no observations before rollout, so β=0, but a large period after rollout, say half of the total cluster size, so α=0.5 (Figure 1(d)). The total cluster size remains the same regardless of α and β, and the remaining observations are distributed equally between the periods within rollout.

In Appendix 1, we derive a design effect for an SWT with these characteristics

(2) $$\begin{array}{c} DE_{\mathrm{SWT}}=\left(1+\left(m-1\right)\rho \right)\frac{3k\left(k-1\right)}{2\left(k+1\right)}\\ \frac{\left(1-R\right)}{\left[1-\left(\beta +\alpha \right)\right]\left[k\left(1-0.5R\left[1-\left(\beta +\alpha \right)\right]\right)-1\right]} \end{array}$$

The terms α and β only affect the design effect through their sum α+β and so it is the combined proportion of observations outside rollout that affects the power, rather than the individual quantities. α and β are also exchangeable in this equation; this means that observations before and after rollout have the same impact on power. This is due to the assumption of observations being equally correlated within each cluster.

### Minimising the sample size of an SWT

In Appendix 2, we show that the optimised SWT has no observations outside rollout (α+β=0; Figure 1(c)) with the number of sequences depending on the ICC and total cluster size, as shown in equation (3)

(3) $$\mathrm{Optimal}\;\mathrm{number}\;\mathrm{of}\;\mathrm{sequences}=\frac{1}{1-\sqrt{R}}$$

Equation (3) will give a non-integer number; to find the exact optimal number of sequences, calculate the design effect (equation (2)) for the integers either side of the result given by equation (3), but a rule of thumb is to round the result to the nearest integer.

**Figure 2.**
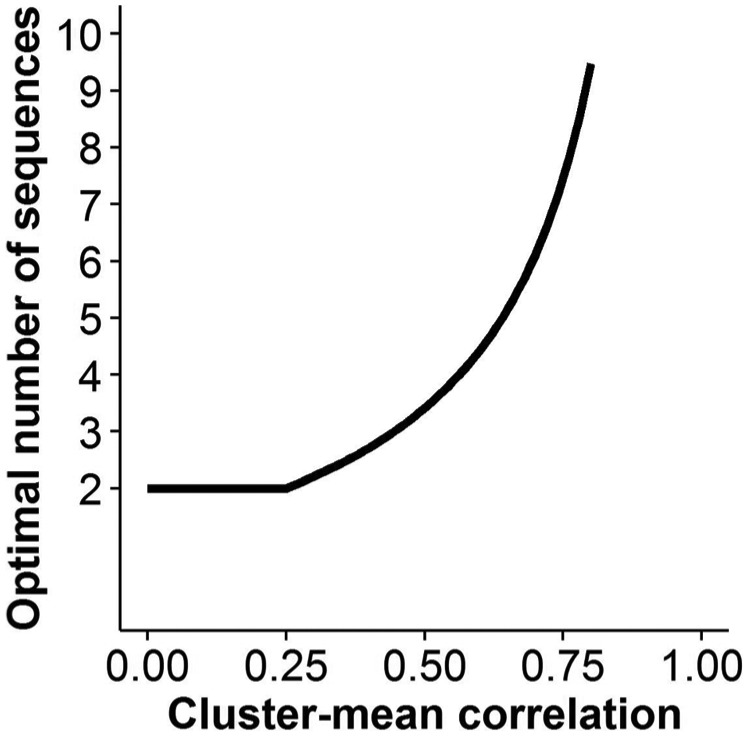
Optimal number of sequences by the cluster-mean correlation. The number of sequences tends to infinity as the cluster-mean correlation tends to 1.

The optimal number of sequences increases as the cluster-mean correlation increases (i.e. the ICC or total cluster size increase), but for low cluster-mean correlation (low ICC or small total cluster size), a small number of sequences is optimal. For example, with 100 observations per cluster and an ICC = 0.01 (*R* = 0.50), it is optimal to have 3 sequences, but with an ICC = 0.1 (*R* = 0.92), it is optimal to have 24 sequences. Figure 2 shows the optimal number of sequences for different cluster-mean correlations.

### Minimising the sample size of a CRT with baseline observations

The design effect (equation (2)) can also give the optimal proportion of baseline observations for a CRT. In Appendix 4, we show that the proportion of observations at baseline that minimises the sample size of a CRT with baseline observations is as follows

(4) $$\beta =\left\{ \begin{array}{cc} 1-\frac{1}{2R} & \mathrm{if}\;R\geq \frac{1}{2}\\ 0 & \mathrm{otherwise} \end{array}\right.$$

For low values of the cluster-mean correlation, it is optimal to have no baseline observations, and for higher values, the optimal proportion of baseline measurements increases to a ceiling of 50% of observations.

### Comparison of an optimised SWT to a CRT

In Appendix 3, we show that when the optimal number of sequences from equation (3) is <2.5, this means that a CRT would require a smaller sample size than any SWT with no observations outside rollout. As a rule of thumb, a CRT will require a smaller sample size when

$$\rho <\frac{1}{\frac{9}{16}m+1}$$

For example, with 100 observations per cluster, a CRT will require fewer clusters than an SWT with no observations outside rollout if ICC < 0.005.

Alternatively, a CRT can be compared to a specific SWT with *k* sequences and no observations outside rollout. The CRT will require a smaller sample size when

$$\rho <\frac{1}{\frac{\left(k+1\right)}{\left(k-1\right)}m+1}$$

In Appendix 5, we show that a CRT with baseline observations will always require the same or a larger sample size than the optimised SWT.

### Comparison to a hybrid design

**Figure 3.**
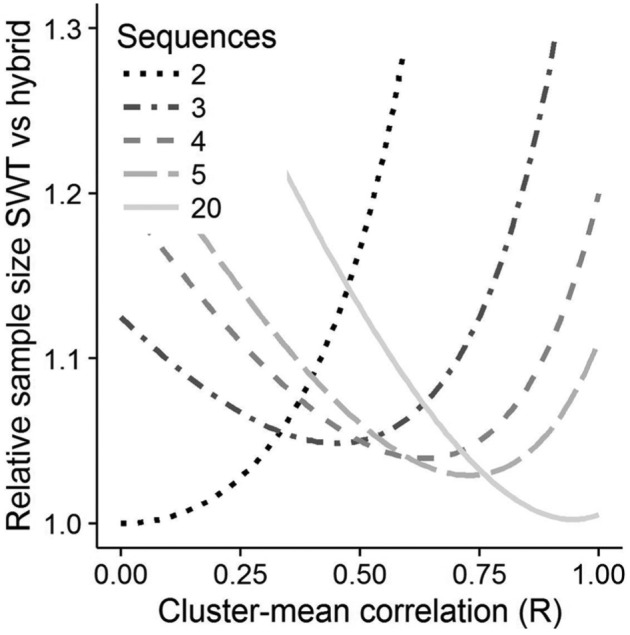
Graph of the sample size of the SWT with no observations outside rollout, relative to the optimised hybrid against the cluster-mean correlation. Darkest and dotted line = 2 sequences, lightest and solid line = 20 sequences. The optimal SWT is the lowest line at any given cluster-mean correlation.

Figure 3 shows the relative sample size of the SWT with no observations outside rollout and 3, 4, 5, or 20 sequences compared to the optimised hybrid design. The optimal SWT, with the optimal number of sequences, is the lowest line at any value of *R*. For example, at *R* = 0.2, two sequences are optimal, but at *R* = 0.7, five sequences are optimal. While the hybrid always has the smaller sample size of the two designs, the differences are small when compared to the optimal SWT, and the optimal SWT requires at most a 5% larger sample size than the hybrid design.

### Other pragmatic SWT designs: a non-optimal number of sequences and including observations outside rollout

It may not always be practical to use the optimal number of sequences calculated in equation (3) as this may be a large number. The primary constraint on the number of sequences is that it cannot exceed the number of clusters in the trial, and the number of periods in the trial cannot exceed the total cluster size. Furthermore, in many settings, the logistical effort to implement the intervention at many different time points would be too great.

In such cases, a smaller, feasible number of sequences could be selected and there may then be some gain from obtaining observations outside rollout. For a fixed number of sequences, the optimal proportion of observations outside rollout (see Appendix 2 for derivation) is a function of the number of sequences and the cluster-mean correlation, as shown in equation (5)

(5) $$\alpha +\beta =\left\{ \begin{array}{cc} 1-\frac{\left(k-1\right)}{k}\frac{1}{R} & \mathrm{if}\;R\geq \frac{\left(k-1\right)}{k}\\ 0 & \mathrm{otherwise} \end{array}\right.$$

For low values of the cluster-mean correlation, that is, low ICC or small total cluster size, it is optimal to have no observations outside rollout, and for higher values, as the ICC or total cluster size increases, the optimal proportion outside rollout increases up to a proportion of 1/*k*.

This proportion varies between 0 (no observations outside rollout) and 1/*k* (equivalent to the same number of observations outside rollout as in one period of the trial). This tells us that the standard SWT, with 1/*k* observations before and 1/*k* observations after rollout, is inefficient.

For an SWT with the proportion of observations outside rollout selected from equation (5), increasing the number of sequences reduces the sample size of the design (see Appendix 7). However, there is little gain from increasing past five sequences, after which there is a maximum 4% further reduction in the sample size. This is only true while R≥(k−1)/k, equivalent to k<1/(1−R). When the number of sequences passes this threshold, the sample size is smallest with no observations outside rollout and increasing the number of sequences will only continue to reduce the sample size up to the optimal number of sequences from equation (3). Appendix 7 contains comparisons of an SWT with this proportion of the observations outside rollout to the other trial designs considered in this article.

### Selecting an SWT design

One strategy for selecting an SWT design with an equal number of clusters in each sequence and an equal number of observations in each period is as follows:

1. Calculate the optimal number of sequences using equation (3).
2. If the number of sequences is feasible, then you have the optimal SWT design by selecting this number of sequences and collecting no observations outside of rollout.
3. If the number of sequences is unfeasibly high, select the number closest to this value that is feasible. Then, compare the cluster-mean correlation to the chosen number of sequences using equation (5) to see whether there is any gain from including observations outside rollout.

Several iterations of designs may be needed to achieve an equal number of clusters in each sequence, varying the numbers of clusters or sequences, so the former is a multiple of the latter. Iterations may also be required to achieve an equal number of observations in each period, varying the total cluster size and number of periods, so the former is a multiple of the latter. Alternatively, the Stata command by Hemming et al. can be used to calculate the power of an unbalanced design.^4^ Once the most appropriate SWT design has been identified, the sample size can be compared with other potential designs such as the CRT and hybrid design if these are feasible.

### Example

Consider a CRT designed to yield 80% power to detect a mean difference of 0.1 in a continuous outcome with a total variance of 1, using a two-sided test at the 5% significance level. The ICC is 0.04, and the total number of observations per cluster is 84. Table 1 shows the number of clusters required to achieve 80% power by several designs. For each design, we give the number of clusters given by the relevant design effect and the number of clusters and power after allowing for an equal number of clusters allocated to each sequence. For the power of some designs, we also made small changes to the total cluster size so that there are an equal number of observations in each period of the trial.

**Table 1.** Illustrative example of the number of clusters required by different designs to achieve 80% power to detect a difference of 0.1 with standard deviation of 1.

Design	Calculated number of clusters	Final design		
		Number of clusters after rounding	Total cluster size^a^	Power (%)
Optimised SWT				
8 sequences, no observations outside rollout	86.1	88	84	81
Other SWT designs				
88 sequences, no observations outside rollout	87.7	88	87	81
8 sequences, 22% outside rollout (standard SWT)	94.0	96	81	80
3 sequences, no observations outside rollout	96.9	99	84	81
3 sequences, optimal outside rollout (14%)	94.2	96	84	81
Other designs				
CRT	161.5	162	84	80
CRT with optimal proportion of observations at baseline (36%)	111.6	112	84	80
Hybrid: 78% 17-sequence SWTs (optimal^b^)	84.8	86: 68 SWTs, 18 CRTs	85	81

CRT: parallel cluster-randomised trial; SWT: stepped wedge trial.

Total cluster size = 84, intracluster correlation coefficient (ICC) = 0.04, 5% significance level and 80% power.

Difference in calculated number of clusters and final number of clusters is due to rounding up and the requirement for an equal number of clusters per sequence.

a For power calculations, the total cluster size had to be varied for some of these designs to allow an equal number of observations in each period of the trial.

b The optimal number of sequences was 68, which gave a calculated number of clusters or 84.7. For 17 sequences, the calculated number is higher, but the final number of clusters required was the same as for 68 sequences and allowed a total cluster size similar to the other designs being considered.

The optimised SWT has eight sequences (and no observations outside rollout). After adjusting the number of clusters to get the same number in each sequence, this design required 88 clusters. Increasing the number of sequences to 88 (one cluster randomised to each sequence) resulted in the design effect giving a larger required number of clusters; this design is an impractical design only given to show that the required number of clusters does not decrease with more sequences. Other SWT designs required between 96 and 99 clusters to achieve 80% power.

A CRT requires almost twice as many clusters as the optimised SWT (162 clusters), and a CRT with baseline observations requires 112 clusters. As expected, the optimised hybrid design, with 78% of clusters randomised to an SWT with 17 sequences, required slightly fewer clusters than the optimised SWT.

## Discussion

We have shown that the sample size of an SWT under equal allocation to sequences can be minimised by collecting all observations within rollout. Unlike the standard SWT, in this optimised SWT, the optimal number of sequences depends on the cluster-mean correlation. We have also provided advice on when to consider other trial designs, acknowledging that a hybrid design will be always slightly more efficient.

Our finding that the most efficient SWT design is to have no observations outside rollout, at least if the resulting optimal number of sequences is also feasible, has not been suggested previously. This optimised SWT may, however, be unacceptable because not all the clusters will receive the intervention during the trial. Trialists may want to include some observations after rollout to avoid a ‘disappointment effect’ in the clusters that would not otherwise receive the intervention. Alternatively, the intervention could still be implemented after data collection has been completed.

We found that there were an optimal number of sequences for minimising the sample size of the SWT with no observations outside rollout. The number was large when the cluster-mean correlation was high (high ICC or large total cluster size) but small when the cluster-mean correlation was low (small ICC and small total cluster size). This contrasts with previous research for the standard SWT which showed that the sample size reduced as the number of sequences increased.^2,3^ It is, however, consistent with the consensus in the literature and finding of this study that a CRT requires a smaller sample size than an SWT when the ICC and total cluster size are low.^7,10^

We examined the optimal proportion of baseline observations in a CRT. We found that when the cluster-mean correlation is low, there is no benefit for the power of the study from including baseline observations. This is because when the ICC is high, the baseline observations will explain more of the variability in the follow-up measurements than when the ICC is low. Our results differed to much of the current literature that suggests that there is always a benefit to including baseline measurements.^14,15^ In this literature, total cluster size was not held constant – instead, baseline observations were included as additional observations relative to a design with no baseline.

This article is the first to compare the sample size implications of increasing the proportion of observations outside rollout versus increasing the number of sequences. We have found that increasing the number of sequences can have a larger impact on the sample size than increasing the proportion of observations outside rollout. For example, there is a larger reduction in sample size (providing the ICC and total cluster size are large enough) going from a CRT to an SWT with three sequences and no observations outside rollout than adding baseline observations to a CRT.

We found that the optimal number of sequences quickly increased with the ICC and total cluster size to a number that may not be practical. In cases such as this where a non-optimal number of sequences is chosen, we found that observations outside rollout may compensate and provide a reduction in the sample size; however, it is never beneficial to the sample size to have more observations outside rollout than are collected in one period of the trial, similar to the results from Girling and Hemming.^7^

Some recently published SWTs included a large proportion of data outside of rollout, usually with the justification of investigating the longer-term effect of the intervention.^1,16^ These designs will give a larger variance for the intervention effect than our optimised SWT design with the same number of observations would have done. Trialists should also be aware that with no control observations after rollout, it will be difficult to assess whether changes in the outcome are due to changes in the intervention effect or other reasons. Our design effect assumes that the intervention effect remains constant throughout the trial. If this is not expected to be the case, different methods of sample size calculation, such as simulations,^5^ and more complex analysis methods should be used.

We found that the hybrid design was more efficient than the optimised SWT, as expected, due to its additional flexibility to allow unequal allocation to sequences. However, the gain in efficiency from this flexibility was at most 5%. Therefore, where considerable additional resources would be required to implement the intervention in a larger number of clusters at the start of the trial than at subsequent switches, the hybrid design will be unattractive. This might be the case if, for example, there is only one team available to roll the intervention out. The optimised hybrid design does not, however, always allocate more clusters to implement the intervention immediately than to other sequences, so one approach to design is to first see whether the optimised hybrid is feasible, and if not, then consider the optimised SWT under equal allocation.

We have given comparisons to some alternative designs, but there are many designs that we have not included. We have not explored incomplete designs such as the dog-leg design or unbalanced SWTs.^17,18^ We have compared trial designs fixing the total cluster size, but a further area of research could vary the total cluster size and fix the number of clusters or look to minimise a combination of the two. In some settings, there may be little or no cost associated with collecting observations before or after rollout, for example, with routinely collected data. If this is the case, it may be more informative to compare trial designs for a given cost rather than a fixed total cluster size.

As with all design effects, the assumptions made about the data must hold for the design effect to be valid, such as exchangeability within clusters and time trends that are common to all clusters. If these assumptions do not hold, using the design effect given here may result in an underpowered trial as the assumed analysis model would be inappropriate. These assumptions have sometimes been criticised as being unrealistic, and others have provided design effects where some assumptions have been relaxed.^19–21^ Baio et al. found the assumption of normality affected sample size calculations for binary outcomes.^5^

Power is only one consideration of many when selecting a trial design. Caution should also be used in designing trials with very few clusters; among other issues, this may reduce generalisability and increase the possibility of chance imbalances.^22^ The lower sample size requirements of SWT and hybrid designs compared to a CRT come at the cost of requiring assumptions about how the outcome is changing over time because the intervention effect is confounded with time. Care needs to be taken to ensure that these assumptions are appropriate and that the analysis takes this into account adequately.^19^

We have identified SWT designs that require fewer clusters than the standard SWT and facilitated comparisons of statistical power between competing trial designs. Following our guidance on selecting a design will result in more efficient trials.

## Supplementary Material

Supplementary material
