# Supplementary material for: The optimal design of stepped wedge trials with equal allocation to sequences and a comparison to other trial designs
Source: Clin Trials. 2017 Aug 10;14(6):639–47. doi: 10.1177/1740774517723921 (PMC5718336; doi:10.1177/1740774517723921)
Supplement: Supplementary material [file 723921_supp_mat.pdf]

Appendix 1:Derivation of the design effect

Throughout this appendix we will use the following notation:

i is the number of clusters that switch at each step,

k is the number of sequences,

z is the number of clusters ($= ik$)

X_{lj} is an idicator variable of whether cluster l is receiving the intervention in period j

b is the multiplier for the number of observations before rollout compared to between steps, so if twice as many are collected before rollout this would be 2.

a is a multipler for how many observations are collected after rollout relative to the number between steps.

β is the proportion of the total cluster size that are before rollout

α is the proportion of the total cluster size that are after rollout

N is the number of observations collected in each cluster between steps.

m is the total cluster size.

ρ is the ICC.

The formula for the variance of the intervention effect from the Hussey and Hughes analysis model [2]:

$$Var\left(\hat{\theta}\right) = \frac{I\sigma^2\left(\sigma^2 + T\tau^2\right)}{\left(IU - W\right)\sigma^2 + \left(U^2 + ITU - TW - IV\right)\tau^2}$$

where σ^2 is the variance of the cluster sample mean in a given period around the true mean, and τ^2 is the between-cluster variance of the cluster means in a given period, and the other terms are defined as follows:

$$\begin{aligned} I &= ik \\ T &= b + (k-1) + a \\ U &= \sum_{lj} X_{lj} \\ W &= \sum_j (\sum_l X_{lj})^2 \\ V &= \sum_l (\sum_j X_{lj})^2 \end{aligned}$$

We want this formula in terms of the total variance σ_t^2, so can use the following substitutions (see [3] for derivations):

$$\sigma^2 = \frac{(1-\rho)\,\sigma_t^2}{N}$$

$$\tau^2 = \rho\sigma_t^2 = \frac{N\rho\sigma_t^2}{N}$$

$$\begin{aligned} Var\left(\hat{\theta}\right) &= \frac{I\frac{(1-\rho)\sigma_t^2}{N}\left(\frac{(1-\rho)\sigma_t^2}{N} + T\frac{N\rho\sigma_t^2}{N}\right)}{(IU-W)\frac{(1-\rho)\sigma_t^2}{N} + (U^2 + ITU - TW - IV)\frac{N\rho\sigma_t^2}{N}} \\ &= \frac{I\,(1-\rho)\,\sigma_t^2}{N}\frac{((1-\rho) + TN\rho)}{(IU-W)\,(1-\rho) + (U^2 + ITU - TW - IV)\,N\rho} \end{aligned}$$

We can work out the component parts to put into this:

$$\begin{aligned} I &= ik \\ T &= b + (k-1) + a \\ U &= \sum_{lj} X_{lj} \\ W &= \sum_j (\sum_l X_{lj})^2 \\ V &= \sum_l (\sum_j X_{lj})^2 \end{aligned}$$

$$
\begin{aligned}
U &= i((1+2+...+(k-1))+ak) \\
&= i(\frac{1}{2}k^2 - \frac{1}{2}k + ak)
\end{aligned}
$$

$$
\begin{aligned}
W &= (1i)^2 + (2i)^2 + ... + ((k-1)i)^2 + a(ki)^2 \\
&= i^2\left(\frac{k^3}{3} - \frac{k^2}{2} + \frac{k}{6} + ak^2\right)
\end{aligned}
$$

$$
\begin{aligned}
V &= i(a)^2 + i(a+1)^2 + i(a+2)^2 + ... + i(a+(k-1))^2 \\
&= i(\frac{k^3}{3} - \frac{k^2}{2} + \frac{k}{6} + a^2k + ak^2 - ak)
\end{aligned}
$$

Now combine these to get the component parts:

$$
\begin{aligned}
IU - W &= ik\left(i\left(\frac{1}{2}k^2 - \frac{1}{2}k + ak\right)\right) - i^2\left(\frac{k^3}{3} - \frac{k^2}{2} + \frac{k}{6} + ak^2\right) \\
&= \frac{i^2}{6}k\left(k^2 - 1\right)
\end{aligned}
$$

$$
\begin{aligned}
U^2 &= i^2k^2(\frac{1}{2}k - \frac{1}{2} + a)^2 \\
&= i^2k\left(\frac{1}{4}k^3 - \frac{1}{2}k^2 + ak^2 - ak + a^2k + \frac{1}{4}k\right)
\end{aligned}
$$

$$
\begin{aligned}
ITU &= ik\left(b+k-1+a\right)\left(i\left(\frac{1}{2}k^2 - \frac{1}{2}k + ak\right)\right) \\
&= i^2k\left(\frac{1}{2}bk^2 - \frac{1}{2}bk + abk + \frac{1}{2}k^3 - k^2 + \frac{1}{2}k + \frac{3}{2}ak^2 - \frac{3}{2}ak + a^2k\right)
\end{aligned}
$$

$$
\begin{aligned}
TW &= (b+k-1+a)\left(i^2\left(\frac{k^3}{3} - \frac{k^2}{2} + \frac{k}{6} + ak^2\right)\right) \\
&= i^2k\left(\frac{1}{3}bk^2 - \frac{1}{2}bk + \frac{1}{6}b + abk + \frac{1}{3}k^3 - \frac{5}{6}k^2 + \frac{4}{6}k - \frac{1}{6} + \frac{4}{3}ak^2 - \frac{3}{2}ak + \frac{1}{6}a + a^2k\right)
\end{aligned}
$$

$$IV = i^2k\left(\frac{k^3}{3} - \frac{k^2}{2} + \frac{k}{6} + a^2k + ak^2 - ak\right)$$

$$\begin{aligned} U^2 - IV &= i^2k\left(\left(\frac{1}{4}k^3 - \frac{1}{2}k^2 + ak^2 - ak + a^2k + \frac{1}{4}k\right) - \left(\frac{k^3}{3} - \frac{k^2}{2} + \frac{k}{6} + a^2k + ak^2 - ak\right)\right) \\ &= i^2k\left(-\frac{1}{12}k^3 + \frac{1}{12}k\right) \end{aligned}$$

$$\begin{aligned} ITU - TW &= i^2k\left(\frac{1}{2}bk^2 - \frac{1}{2}bk + abk + \frac{1}{2}k^3 - k^2 + \frac{1}{2}k + \frac{3}{2}ak^2 - \frac{3}{2}ak + a^2k\right) \\ &\quad -i^2k\left(\frac{1}{3}bk^2 - \frac{1}{2}bk + \frac{1}{6}b + abk + \frac{1}{3}k^3 - \frac{5}{6}k^2 + \frac{4}{6}k - \frac{1}{6} + \frac{4}{3}ak^2 - \frac{3}{2}ak + \frac{1}{6}a + a^2k\right) \\ &= i^2k\left(\frac{1}{6}bk^2 - \frac{1}{6}b + \frac{1}{6}k^3 - \frac{1}{6}k^2 - \frac{1}{6}k + \frac{1}{6} + \frac{1}{6}ak^2 - \frac{1}{6}a\right) \end{aligned}$$

$$\begin{aligned} U^2 + ITU - TW - IV &= i^2k\left(-\frac{1}{12}k^3 + \frac{1}{12}k + \frac{1}{6}bk^2 - \frac{1}{6}b + \frac{1}{6}k^3 - \frac{1}{6}k^2 - \frac{1}{6}k + \frac{1}{6} + \frac{1}{6}ak^2 - \frac{1}{6}a\right) \\ &= \frac{i^2k\left(k^2-1\right)}{6}\left(b + \frac{1}{2}k - 1 + a\right) \end{aligned}$$

Substituting this into the variance formula gives:

$$\begin{aligned} Var\left(\hat{\theta}\right) &= \frac{I\left(1-\rho\right)\sigma_t^2}{N}\frac{\left(\left(1-\rho\right) + TN\rho\right)}{\left(IU - W\right)\left(1-\rho\right) + \left(U^2 + ITU - TW - IV\right)N\rho} \\ &= \frac{ik\left(1-\rho\right)\sigma_t^2}{N}\frac{\left(\left(1-\rho\right) + \left(b+k-1+a\right)N\rho\right)}{\left(\frac{i^2}{6}k\left(k^2-1\right)\right)\left(1-\rho\right) + \left(\frac{i^2}{6}k\left(k^2-1\right)\left(b+\frac{1}{2}k-1+a\right)\right)N\rho} \\ &= \frac{\left(1+\rho\left(bN+kN+aN-N-1\right)\right)}{\left(1+\rho\left(bN+\frac{1}{2}kN+aN-N-1\right)\right)}\frac{6\left(1-\rho\right)}{\left(k-\frac{1}{k}\right)}\frac{\sigma_t^2}{Nik} \end{aligned}$$

To give this formula in terms of the total cluster size m, and proportions before and after rollout β and α respectively, we can use the following substitutions:

$$\begin{aligned} m &= bN + Nk - N + aN \\ \beta &= \frac{bN}{m} \\ \alpha &= \frac{aN}{m} \end{aligned}$$

So the variance becomes

$$\begin{aligned}
Var\left(\hat{\theta}\right) &= 6\sigma_t^2 \frac{(1+\rho(m-1))}{\left(1+\rho\left(m-1-\frac{1}{2}kN\right)\right)} \frac{(1-\rho)}{\left(k-\frac{1}{k}\right)} \frac{1}{Nik} \\
&= 6\sigma_t^2 \frac{(1+\rho(m-1))}{\left(1+\rho\left(m-1-\frac{1}{2}kN\right)\right)} \frac{(1-\rho)}{\left(k-\frac{1}{k}\right)} \frac{1}{z\frac{m(1-\beta-\alpha)}{(k-1)}} \\
&= 6\sigma_t^2 \frac{(1+\rho(m-1))}{\left(1+\rho\left(m-1-\frac{1}{2}kN\right)\right)} \frac{(1-\rho)}{\left(k-\frac{1}{k}\right)} \frac{k-1}{zm(1-\beta-a)} \\
&= \frac{6\sigma_t^2}{mz}(1+\rho(m-1))(1-\rho)\frac{k}{(k+1)} \frac{1}{(1-\beta-a)\left(1+(m-1)\rho-\frac{1}{2}\frac{k}{k-1}(1-\beta-\alpha)\rho m\right)} \quad (0.0.1)
\end{aligned}$$

The variance of a trial with this sample size if it was individually randomised would be

$$Var(\theta_{ind}) = \frac{4\sigma_t^2}{mz}$$

So our design effect becomes:

$$DE \quad = \frac{3}{2} \quad (1+\rho(m-1))(1-\rho)\frac{k}{(k+1)} \frac{1}{(1-\beta-\alpha)\left(1+(m-1)\rho-\frac{1}{2}\frac{k}{k-1}(1-\beta-\alpha)\rho m\right)}$$

We can rewrite this in terms of the cluster mean correlation defined by Girling and Hemming [1]:

$$R = \frac{\rho m}{(1+\rho(m-1))}$$

Our design effect becomes:

$$DE \quad = \frac{3}{2} \quad (1+\rho(m-1))\frac{k(k-1)}{(k+1)} \frac{(1-R)}{(1-\beta-\alpha)(k(1-0.5R(1-\beta-\alpha))-1)}$$

In this paper we discuss minimising the design effect in terms of the number of sequences, k, and the proportion outside of rollout $\beta+\alpha$. Note that to do this only the last 2 terms of the DE are manipulated. All other parts of the design effect are held constant. Also note that these last 2 terms are the same as the last 2 terms in the variance formula eq (0.0.1). So minimising the design effect with respect to k and $\alpha+\beta$ is equivalent to minimising the variance. We can also rearrange eq (0.0.1) to give a formula for the number of clusters, these last 2 terms remain the same with all other terms remaining constant regardless of the values of k and $\beta+\alpha$. This means that the results we find for the values of k and $\beta+\alpha$ to minimse the design effect give the optimal values to minimise the number of clusters, or the variance.

Appendix 2: Optimal number of sequences and optimal proportion of observations outside rollout

We want to find the combination of number of sequences k and the proportion outside rollout, $\alpha+\beta$, that minimises the design effect. We can do this by partially differentiating the design effect, firstly in terms of the number of sequences to get an equation for the optimal k, and secondly in terms of $\alpha+\beta$ to get an equation for the optimal $\alpha+\beta$. We will then solve these 2 equations simultaneously to find an optimum design for a given m and ρ.

We have boundaries on these values so we also need to check for optimal values at the boundaries. The boundaries are:

$$k \geq 2$$

$$0 \leq \alpha+\beta \leq 1$$

Let $\delta = \alpha+\beta$ be the proportion of observations that are outside rollout. Substituting this into the design effect gives:

$$DE = (1+\rho(m-1))\frac{3k}{2(k+1)}\frac{(1-\rho)}{(1-\delta)\left(1+(m-1)\rho-\frac{1}{2}\frac{k}{k-1}(1-\delta)\rho m\right)}$$

Optimising the number of sequences k

Although k will be an integer, we will treat it as continuous and assume that the optimal k will be one of the integers either side of the idenitified continuous optimal value. This means that we can differentiate the design effect with respect to k to get an equation for the optimal number or sequences for a given δ:

$$
\begin{aligned}
DE &= (1+\rho(m-1))\frac{3k}{2(k+1)}\frac{(1-\rho)}{(1-\delta)\left(1+(m-1)\rho-\frac{1}{2}\frac{k}{k-1}(1-\delta)\rho m\right)} \\
\frac{d(DE)}{dk} &= \frac{3(1+\rho(m-1))(1-\rho)}{2(1-\delta)}\frac{d}{dk}\left(\frac{k}{(k+1)\left(1+(m-1)\rho-\frac{1}{2}\frac{k}{k-1}(1-\delta)\rho m\right)}\right) \\
&= \frac{3(1+\rho(m-1))(1-\rho)}{2(1-\delta)}\frac{k^2(1+\rho(m\delta-1))-2k(1+\rho(m-1))+(1+\rho(m-1))}{\left((k^2-1)(1+\rho(m-1))-\frac{1}{2}(1-\delta)\rho mk(k+1)\right)^2}
\end{aligned}
$$

The optimal number of sequences is when the derivative is equal to zero. So

$$
k^2(1+\rho(m\delta-1))-2k(1+\rho(m-1))+(1+\rho(m-1))=0
$$

$$
\begin{aligned}
k &= \frac{2(1+\rho(m-1))\pm\sqrt{(2(1+\rho(m-1)))^2-4(1+\rho(m\delta-1))(1+\rho(m-1))}}{2(1+\rho(m\delta-1))} \\
&= \frac{(1+\rho(m-1))\pm\sqrt{(1-\delta)\rho m(1+\rho(m-1))}}{(1+\rho(m\delta-1))}
\end{aligned}
$$

There are 2 solutions here. The negative square root will only give a number of sequences greater than 2 when

$$
\rho<\frac{1}{(3m+1)}
$$

This suggests that the negative square root does not give the minimum. Graphical inspection and numerical example support that the positive square root gives a minimum value of the design effect. So the optimal number of sequences fixing all other parameters is

$$
k=\frac{(1+\rho(m-1))+\sqrt{(1-\delta)\rho m(1+\rho(m-1))}}{(1+\rho(m\delta-1))}
$$

Optimising the proportion outside rollout $\alpha+\beta$

We want to find the minimum of the DE with respect to α and β.

Let $\delta=\alpha+\beta$ and differentiate with respect to δ

$$\begin{aligned}\frac{d(DE)}{d\delta} &= (1+\rho(m-1))(1-\rho)\frac{3k}{2(k+1)}\frac{d}{d\delta}\left(\frac{1}{(1-\delta)\left((1+\rho(m-1))-\frac{1}{2}\frac{k}{k-1}\rho m(1-\delta)\right)}\right)\\ &= (1+\rho(m-1))(1-\rho)\frac{3k}{2(k+1)}\left(\frac{(1+\rho(m-1))-\frac{k}{k-1}\rho m(1-\delta)}{(1-\delta)^2\left((1+\rho(m-1))-\frac{1}{2}\frac{k}{k-1}\rho m(1-\delta)\right)^2}\right)\end{aligned}$$

The turning point is when

$$\begin{aligned}(1+\rho(m-1))(1-\rho)\frac{3k}{2(k+1)}\left(\frac{(1+\rho(m-1))-\frac{k}{k-1}\rho m(1-\delta)}{(1-\delta)^2\left((1+\rho(m-1))-\frac{1}{2}\frac{k}{k-1}\rho m(1-\delta)\right)^2}\right) &= 0\\ (1+\rho(m-1))-\frac{k}{k-1}\rho m(1-\delta) &= 0\\ \delta &= 1-\frac{(k-1)(1+\rho(m-1))}{\rho mk}\end{aligned}$$

Taking the second derivative shows that this is a minimum.

δ can get infinitely small but this is not possible under our constraint of $\delta \geq 0$ so we must limit this value to 0 for any value smaller than 0. This happens when:

$$\frac{(k-1)}{k}\frac{(1+\rho(m-1))}{\rho m} > 1$$

Or equivalently

$$\frac{\rho m}{(1+\rho(m-1))} < \frac{k-1}{k}$$

We can write this as the proportion outside rollout fixing all other parameters:

$$\delta = \alpha+\beta = \begin{cases} 1-\frac{(k-1)}{k}\frac{(1+\rho(m-1))}{\rho m}, & \frac{\rho m}{(1+\rho(m-1))} \geq \frac{k-1}{k} \\ 0 & otherwise \end{cases}$$

Since$1 < \frac{(1+\rho(m-1))}{\rho m} < \infty$, this optimal$\alpha+\beta$ has boundaries:

$$0 \leq \alpha+\beta \leq \frac{1}{k}$$

This can also be written in terms of the cluster mean correlation R:

$$\delta = \alpha+\beta = \begin{cases} 1-\frac{(k-1)}{k}\frac{1}{R}, & R \geq \frac{k-1}{k} \\ 0 & otherwise \end{cases}$$

Solving the equations

We now have the simultaneous equations

$$\begin{aligned}\delta &= 1-\frac{(k-1)\,(1+\rho\,(m-1))}{\rho m k}\\ k &= \frac{(1+\rho\,(m-1))+\sqrt{(1-\delta)\,\rho m\,(1+\rho\,(m-1))}}{(1+\rho\,(m\delta-1))}\end{aligned}$$

There is no solution to these simultaneous eqations. This means that there is no minimum value within the boundaries. We must also check for optimal values at the boundaries of k and δ.

Boundary (1) $\delta = 0$

We have a boundary at $\delta = 0$, with all observations during rollout.

The equation for optimal k becomes:

$$k = \frac{(1+\rho\,(m-1))+\sqrt{\rho m\,(1+\rho\,(m-1))}}{(1-\rho)}$$

Substituting this k and $\delta = 0$ into the design effect we get:

$$DE_{(1)} = 3\frac{\left(2\rho m\,(1+\rho\,(m-1))+(1+\rho\,(2m-1))\,\sqrt{\rho m\,(1+\rho\,(m-1))}\right)(1-\rho)\,(1+\rho\,(m-1))}{\left((2+\rho\,(m-2))+\sqrt{\rho m\,(1+\rho\,(m-1))}\right)\left(\rho m\,(1+\rho\,(m-1))+(2+\rho\,(m-2))\,\sqrt{\rho m\,(1+\rho\,(m-1))}\right)}$$

Boundary (2) $\delta = 1/k$

Although $0 < \delta < 1$, in our derivation of the optimal value of δ, we found that this had a boundary at $\delta = 1/k$

The design effect in this case is:

$$DE_{(2)} = (1+\rho(m-1))\frac{3k^2}{2(k^2-1)}\frac{(1-\rho)}{((1+\rho(0.5m-1)))}$$

$\frac{k^2}{(k^2-1)}$ decreases as k increases so at this boundary the optimal design is to have a large number of sequences. The design effect here becomes:

$$DE = \frac{3(1-\rho)(1+\rho(m-1))}{(2+\rho(m-2))}$$

Boundary (3) $k = 2$

The final boundary to the design effect is the lower limit of k. The optimal value of δ when $k = 2$ is:

$$\delta = \begin{cases} 1 - \frac{1}{2}\frac{(1+\rho(m-1))}{\rho m}, & \frac{\rho m}{(1+\rho(m-1))} \geq \frac{1}{2} \\ 0 & otherwise \end{cases}$$

With these values the design effect becomes:

$$DE_{(3)} = \begin{cases} \frac{4\rho m(1-\rho)}{(1+\rho(m-1))}, & \frac{\rho m}{(1+\rho(m-1))} \geq \frac{1}{2} \\ (1+\rho(m-1)) & otherwise \end{cases}$$

Which boundary is optimal?

We can work out which of these is optimal by looking at the ratio of these design effects.

$\delta = 0$ vs $\delta = 1/k$

Taking the ratio of the design effects when $\delta = 0$ and when $\delta = 1/k$ gives

$$\begin{aligned}\frac{DE_{(2)}}{DE_{(1)}} &= \left(\frac{3\left(2\rho m(1+\rho(m-1)) + (1+\rho(2m-1))\sqrt{\rho m(1+\rho(m-1))}\right)(1-\rho)(1+\rho(m-1))}{\left((2+\rho(m-2)) + \sqrt{\rho m(1+\rho(m-1))}\right)\left(\rho m(1+\rho(m-1)) + (2+\rho(m-2))\sqrt{\rho m(1+\rho(m-1))}\right)}\right) \\ &\quad * \left(\frac{(2+\rho(m-2))}{3(1-\rho)(1+\rho(m-1))}\right) \\ &= \frac{\left(2\rho m(1+\rho(m-1)) + (1+\rho(2m-1))\sqrt{\rho m(1+\rho(m-1))}\right)(2+\rho(m-2))}{\left((2+\rho(m-2)) + \sqrt{\rho m(1+\rho(m-1))}\right)\left(\rho m(1+\rho(m-1)) + (2+\rho(m-2))\sqrt{\rho m(1+\rho(m-1))}\right)}\end{aligned}$$

We are interested in when this ratio is less than 1:

$$\frac{\left(2\rho m\left(1+\rho\left(m-1\right)\right)+\left(1+\rho\left(2m-1\right)\right)\sqrt{\rho m\left(1+\rho\left(m-1\right)\right)}\right)\left(2+\rho\left(m-2\right)\right)}{\left(\left(2+\rho\left(m-2\right)\right)+\sqrt{\rho m\left(1+\rho\left(m-1\right)\right)}\right)\left(\rho m\left(1+\rho\left(m-1\right)\right)+\left(2+\rho\left(m-2\right)\right)\sqrt{\rho m\left(1+\rho\left(m-1\right)\right)}\right)} \quad < \quad 1$$

Using Mathematica software shows that this inequality is true for all $0<\rho<1$, $m>0$.

This means that the design with no observations outside rollout and the optimal number of sequences for that design, will always be more efficient than the design with $1/k$ observations outside rollout and the optimal number of sequences for that design.

$\delta=0$ and optimal k vs $k=2$and optimal δ

To compare the design on the $\delta=0$ boundary to the design on the $k=2$ boundary we need to split the comparison.

When $\frac{\rho m}{(1+\rho(m-1))}\geq\frac{1}{2}$ so when $\rho>\frac{1}{(m+1)}$, we can look at the ratio of design effects for these 2 designs which gives:

$$\begin{aligned}
\frac{DE_{(3a)}}{DE_{(1)}} &= \left(\frac{4\rho m\left(1-\rho\right)}{\left(1+\rho\left(m-1\right)\right)}\right) \\
&\quad *\left(\frac{\left(\left(2+\rho\left(m-2\right)\right)+\sqrt{\rho m\left(1+\rho\left(m-1\right)\right)}\right)\left(\rho m\left(1+\rho\left(m-1\right)\right)+\left(2+\rho\left(m-2\right)\right)\sqrt{\rho m\left(1+\rho\left(m-1\right)\right)}\right)}{3\left(2\rho m\left(1+\rho\left(m-1\right)\right)+\left(1+\rho\left(2m-1\right)\right)\sqrt{\rho m\left(1+\rho\left(m-1\right)\right)}\right)\left(1-\rho\right)\left(1+\rho\left(m-1\right)\right)}\right) \\
&= \frac{4\rho m\left(\left(2+\rho\left(m-2\right)\right)+\sqrt{\rho m\left(1+\rho\left(m-1\right)\right)}\right)\left(\rho m\left(1+\rho\left(m-1\right)\right)+\left(2+\rho\left(m-2\right)\right)\sqrt{\rho m\left(1+\rho\left(m-1\right)\right)}\right)}{3\left(2\rho m\left(1+\rho\left(m-1\right)\right)+\left(1+\rho\left(2m-1\right)\right)\sqrt{\rho m\left(1+\rho\left(m-1\right)\right)}\right)\left(1+\rho\left(m-1\right)\right)^2}
\end{aligned}$$

We can find out when this ratio of design effects is greater than 1, i.e. when the design effect at $k=2$ optimal δ is smaller that the design effect at optimal k and $\delta=0$:

$$\frac{4\rho m\left(\left(2+\rho\left(m-2\right)\right)+\sqrt{\rho m\left(1+\rho\left(m-1\right)\right)}\right)\left(\rho m\left(1+\rho\left(m-1\right)\right)+\left(2+\rho\left(m-2\right)\right)\sqrt{\rho m\left(1+\rho\left(m-1\right)\right)}\right)}{3\left(2\rho m\left(1+\rho\left(m-1\right)\right)+\left(1+\rho\left(2m-1\right)\right)\sqrt{\rho m\left(1+\rho\left(m-1\right)\right)}\right)\left(1+\rho\left(m-1\right)\right)^2} \quad > \quad 1$$

Rearranging gives:

$$\begin{aligned}
& 2\rho m\left(1+\rho\left(m-1\right)\right)\left(\rho^2\left(m-3\right)\left(m+1\right)+2\rho\left(m+3\right)-3\right) \\
& +\left(\rho^3\left(2m^3-5m^2+4m+3\right)+\rho^2\left(5m^2-8m-9\right)+\rho\left(4m+9\right)-3\right)\sqrt{\rho m\left(1+\rho\left(m-1\right)\right)} \quad > \quad 0
\end{aligned}$$

If each component of the sum is > 0, then there sum will be > 0 and the inequality will hold.

Using Mathematica software shows that

$$\left(\rho^2 (m-3)(m+1) + 2\rho (m+3) - 3\right) > 0$$

when $\rho > 1/(m+1)$. This is multiplied by 2 positive numbers so this term will be positive if $\rho > 1/(m+1)$.

Secondly,

$$\left(\rho^3 \left(2m^3 - 5m^2 + 4m + 3\right) + \rho^2 \left(5m^2 - 8m - 9\right) + \rho (4m+9) - 3\right) > 0$$

when $\rho > 1/(m+1)$. Since the square root term is also postive, this whole second term will be positive when $\rho > 1/(m+1)$.

So all terms are positive and so the total is postive when $\rho > 1/(m+1)$. This means that, under this conditions, the design with $\delta = 0$ has the smaller design effect.

But, we are currently looking in the region where $\frac{\rho m}{(1+\rho(m-1))} \geq \frac{1}{2}$. In this region$\rho > 1/(m+1)$.

This means that in the region where $\frac{\rho m}{(1+\rho(m-1))} \geq \frac{1}{2}$, $DE_{(3)} > DE_{(1)}$.

Now looking at when $\frac{\rho m}{(1+\rho(m-1))} < \frac{1}{2}$.

In this region both designs have $\delta = 0$ so we are comparing a design with $\delta = 0$ and an optimal number of sequences that can equal 2 if that is optimal, and a design with the number of sequences fixed to 2 and $\delta = 0$. By definition the design when the number of sequences is allowed to vary will be either the same or more efficient than the design where the number of sequences is fixed to 2.

Conclusion

We can conclude that the optimal stepped wedge design is to have $\delta = 0$, or equivalently $\alpha + \beta = 0$, and k as:

$$k = \frac{(1 + \rho (m-1)) + \sqrt{\rho m (1 + \rho (m-1))}}{(1-\rho)}$$

The equation for k becomes:

$$k = \frac{1}{1 - \sqrt{R}}$$

Appendix 3 SWT with no observations outside rollout compared to a CRT

We will compare an SWT with no observations outside rollout to a CRT in 2 ways. Firstly we can compare the ratio of the design effects of a CRT to a specific SWT design. Secondly we can investigate when the optimal number of sequences in the SWT is < 2.5 so that the SWT becomes equivalent to a CRT.

Comparison of design effects

DE of SWT:

$$DE = (1 + \rho (m-1)) \frac{3k}{2(k+1)} \frac{(1-\rho)}{\left((1 + \rho (m-1)) - \frac{1}{2}\frac{k}{k-1}\rho m\right)}$$

DE of CRT

$$DE = (1 + \rho (m-1))$$

Note that when $k = 2$ the SWT DE cancels to the CRT DE as with no observations outside rollout and $k = 2$ the design is a CRT.

The ratio of the DEs is

$$\frac{DE_{SWT}}{DE_{CRT}} = \frac{3k}{2(k+1)} \frac{(1-\rho)}{\left(1 + \left(m\left(1 - \frac{1}{2}\frac{k}{k-1}\right) - 1\right)\rho\right)}$$

We are interested in when this ratio is less than 1, so the SWT design effect is smaller than the CRT design effect.

$$
\begin{aligned}
\frac{3k}{2(k+1)} \frac{(1-\rho)}{\left(1+\left(m\left(1-\frac{1}{2}\frac{k}{k-1}\right)-1\right)\rho\right)} &< 1 \\
\frac{3k(k-1)(1-\rho)}{(k-1+(m(0.5k-1)-(k-1))\rho)} &< 2(k+1)
\end{aligned}
$$

Before multiplying up the LHS denominator we need to check when it is positive:

$$
(k-1+(m(0.5k-1)-(k-1))\rho) > 0
$$

This is true when:

$$
k > \frac{1+\rho(m-1)}{1+\rho(0.5m-1)}
$$

$1 < \frac{1+\rho(m-1)}{1+\rho(0.5m-1)} < 2$ because $0 < \rho < 1$ and $m > 1$.

So the LHS denomination is positive for all $k > 2$ so we can mutliple the LHS denominator to both sides of the inequality:

$$
\begin{aligned}
3k(k-1)(1-\rho) &< 2(k+1)(k-1+(m(0.5k-1)-(k-1))\rho) \\
(k-1)(k-2) &< \rho(m(k+1)(k-2)+(k-1)(k-2)))
\end{aligned}
$$

Since $k > 2$ we can divide through by $(k-2)$

$$
\begin{aligned}
(k-1) &< \rho(m(k+1)+(k-1)) \\
\rho &> \frac{1}{\frac{(k+1)}{(k-1)}m+1}
\end{aligned}
$$

Optimal number of sequences is 2

Another way of doing this is to look at when the optimal number of sequences for the SWT is ≥ 2.5. Note that is only approximately true, the function is not symmetrical so if the optimal number of sequences is 2.5, 2 sequences

may be more efficient than 3 sequences in some cases. It should be right for the majority of cases, or good as a rule of thumb.

$$optimal\ k = \frac{(1+\rho(m-1)) + \sqrt{\rho m(1+\rho(m-1))}}{(1-\rho)}$$

$$\begin{aligned}
\frac{(1+\rho(m-1)) + \sqrt{\rho m(1+\rho(m-1))}}{(1-\rho)} &\geq 2.5 \\
\sqrt{\rho m(1+\rho(m-1))} &\geq 2.5(1-\rho) - (1+\rho(m-1))
\end{aligned}$$

In order to square the LHS we need to know whether both sides are positive. $\rho m(1+\rho(m-1)) > 0$ for all values but the RHS could be positive or negative.

If RHS is negative we cannot square both sides but the inequality will still hold. This is the case when

$$\begin{aligned}
2.5(1-\rho) - (1+\rho(m-1)) &\leq 0 \\
\rho &\geq \frac{3}{2m+3} = \frac{9}{6m+9}
\end{aligned}$$

Otherwise, if the RHS is postive, i.e.

$$\rho < \frac{3}{2m+3} = \frac{9}{6m+9}$$

we can square both sides:

$$\begin{aligned}
\rho m(1+\rho(m-1)) &\geq \left(\frac{5}{2}(1-\rho) - (1+\rho(m-1))\right)^2 \\
0 &\geq \rho^2(16m+9) - \rho(16m+18) + 9
\end{aligned}$$

$$\begin{aligned}
\rho &= \frac{-b \pm \sqrt{b^2-4ac}}{2a} \\
a &= (16m+9) \\
b &= -(16m+18) \\
c &= 9 \\
\rho &= \frac{(16m+18) \pm \sqrt{(16m+18)^2 - 4*9(16m+9)}}{2(16m+9)} \\
&= 1\ OR\ \frac{9}{16m+9}
\end{aligned}$$

So we have $(\rho - 1)\left(\rho - \frac{9}{16m+9}\right) \leq 0$. Since $\rho < 1$ we are left with

$$\rho \geq \frac{9}{16m+9}$$

So our 2 solutions combine to show that the optimal number of sequences in the SWT is greater than 2.5 when

$$\rho \geq \frac{9}{16m+9}$$

Appendix 4 Optimal proportion of observations at baseline in a CRT

Our results from Appendix A boundary (3) give the optimal proportion of observations outside rollout for a CRT:

$$\beta = \begin{cases} 1 - \frac{1}{2}\frac{(1+\rho(m-1))}{\rho m}, & \frac{\rho m}{(1+\rho(m-1))} \geq \frac{1}{2} \\ 0 & otherwise \end{cases}$$

In terms of the cluster-mean correlation this is:

$$\beta = \begin{cases} 1 - \frac{1}{2R}, & R \geq \frac{1}{2} \\ 0 & otherwise \end{cases}$$

Appendix 5 Comparison between optimal SWT and a CRT with baseline measurements

This comparison has been seen in Appendix 2, "Which boundary is optimal? $\delta = 0$ vs $k = 2$". This was a comparison of an SWT with $\delta = 0$ and an optimised number of sequences (the optimal SWT design), and an SWT with $k = 2$ (or a CRT with and without baseline observations). We saw that in the region where baseline observations would be beneficial, i.e. when

$$R = \frac{\rho m}{(1 + \rho (m - 1))} \geq \frac{1}{2}$$

the optimised SWT design effect was always smaller than the design effect of the CRT with baseline observations.

Appendix 6 Comparison between optimal SWT and the hybrid design

Comparison with an SWT with no observations outside rollout

Girling and Hemming 2016 [1]define a hybrid design, which consists of a a proprotion of the clusters contributing to the design as an SWT with $\delta = \alpha + \beta = 1/k$, and the remaining clusters contributing as a parallel CRT. We will compare this design, with a large number of sequences in the SWT and an optimal proportion allocated to the SWT, to an SWT with $\delta = \alpha + \beta = 0$ and k sequences.

Let us define γ as the proportion of c.usters assigned to the SWT.

The design effect for the hybrid design is [1]:

$$DE_H = \frac{\eta}{4\left(a_D - b_D R\right)}$$

where

$$\begin{aligned}
\eta &= 1 - \rho = \left(1 + \rho\left(m-1\right)\right)\left(1 - R\right) \\
4a_D &= 1 - \frac{\gamma^2}{3}\left(1 + \frac{2}{k^2}\right) \\
4b_D &= 1 - \frac{\gamma}{3}\left(2 + \frac{1}{k^2}\right) \\
R &= \frac{\rho m}{1 + \rho\left(m-1\right)}
\end{aligned}$$

$$DE_H = \frac{\left(1 + \rho\left(m-1\right)\right)\left(1 - R\right)}{1 - \frac{\gamma^2}{3}\left(1 + \frac{2}{k^2}\right) - \left(1 - \frac{\gamma}{3}\left(2 + \frac{1}{k^2}\right)\right) R}$$

For the optimal Hybrid design, $\gamma = R$ and there are many sequences so that $1/k^2 \approx 0$. The design effect becomes

$$DE_H = \frac{3\left(1+\rho\left(m-1\right)\right)\left(1-R\right)}{3-3R+R^2}$$

Comparing this to our SWT with no opservations outside rollout ($\delta = 0$) and k sequences gives:

$$\begin{aligned}\frac{DE_S}{DE_H} &= \left(1+\rho\left(m-1\right)\right)\frac{3k\left(k-1\right)}{2\left(k+1\right)}\frac{\left(1-R\right)}{\left(k\left(1-0.5R\right)-1\right)}\frac{3-3R+R^2}{3\left(1+\rho\left(m-1\right)\right)\left(1-R\right)} \\ &= \frac{k\left(k-1\right)\left(3-3R+R^2\right)}{2\left(k+1\right)\left(k\left(1-0.5R\right)-1\right)}\end{aligned}$$

This is the function that is shown graphically in figure 3.

Appendix 7 SWT with observations outside rollout

In appendix 2 we showed that for a a fixed number of sequences k, the optimal proportion of observations outside rollout is:

$$\alpha+\beta=\begin{cases}1-\frac{(k-1)}{k}\frac{(1+\rho(m-1))}{\rho m}, & \frac{\rho m}{(1+\rho(m-1))}\geq\frac{k-1}{k}\\ 0 & otherwise\end{cases}$$

or in terms of R:

$$\alpha+\beta=\begin{cases}1-\frac{(k-1)}{k}\frac{1}{R}, & R\geq\frac{k-1}{k}\\ 0 & otherwise\end{cases}$$

Substituting this into the design effect gives:

$$DE=\begin{cases}\frac{3k^2}{(k^2-1)}\frac{\rho m(1-\rho)}{(1+\rho(m-1))}, & \frac{\rho m}{(1+\rho(m-1))}\geq\frac{k-1}{k}\\ (1+\rho\,(m-1))\,\frac{3k}{2(k+1)}\frac{(1-\rho)}{\left(1+(m-1)\rho-\frac{1}{2}\frac{k}{k-1}\rho m\right)} & otherwise\end{cases}$$

or in terms of R:

$$DE=\begin{cases}(1+\rho\,(m-1))\,\frac{3k^2}{(k^2-1)}R\,(1-R)\,, & R\geq\frac{k-1}{k}\\ (1+\rho\,(m-1))\,\frac{3k(k-1)}{2(k+1)}\frac{(1-R)}{(k(1-0.5R)-1)} & otherwise\end{cases}$$

Impact on sample size of increasing the number of sequences in an SWT with the optimal proportion of observations outside rollout\,

While $\frac{\rho m}{(1+\rho(m-1))} \geq \frac{k-1}{k}$ the number of sequences affects to the design effect by a factor of

$$\frac{k^2}{(k^2-1)}$$

This factor reduces as the number of sequences increases as show in the table below:

		Increased number of sequences						
		2	3	4	5	6	7	8
Original number of sequences	2	1.00	0.84	0.80	0.78	0.77	0.77	0.76
	3		1.00	0.95	0.93	0.91	0.91	0.90
	4			1.00	0.98	0.96	0.96	0.95
	5				1.00	0.99	0.98	0.99
	6					1.00	0.99	0.99
	7						1.00	1.00
	8							1.00

The maximum relative difference in sample size after 5 sequences is

$$\frac{1}{\frac{5^2}{(5^2-1)}} = \frac{\left(5^2-1\right)}{5^2} = 0.96$$

Comparison with a CRT with baseline observations

We have previously stated that a CRT with baseline observations can be thought of as an SWT with 2 sequences. Since the design effect decreases as the number of sequences increase a CRT with baseline observations (or equivalently an SWT with 2 sequences and the optimal proportion outside rollout) will always require a larger sample size than an SWT with more sequences and an optimal proportion or observations outside rollout.

Comparison with a CRT

When $\frac{\rho m}{(1+\rho(m-1))} \geq \frac{k-1}{k}$ an SWT with k sequences may benefit from some observations being collected outside rollout. Within this region an SWT with k sequences and the optimal proportion of observations outside rollout will always have a smaller sample size than a CRT. We can show this by comparing the design effect from Appendix 7 to the design effect for a CRT:

$$\frac{DE_{SWT}}{DE_{CRT}} = \frac{3k^2}{(k^2-1)} \frac{\rho m (1-\rho)}{(1+\rho(m-1))} \frac{1}{(1+\rho(m-1))}$$

We are interested in when the design effect of the SWT is smaller than the design effect of the CRT. When this is the case this ratio will be less than 1 so we can look at if and when this is true:

$$\frac{3k^2}{(k^2-1)} \frac{\rho m (1-\rho)}{(1+\rho(m-1))} \frac{1}{(1+\rho(m-1))} \quad < \quad 1$$

Using Mathematica software shows that this is true for all $0 < \rho < 1$, $m > 0$, $and\, k > 2$.

So when $\frac{\rho m}{(1+\rho(m-1))} \geq \frac{k-1}{k}$ the SWT is more efficient than the CRT.

When $\frac{\rho m}{(1+\rho(m-1))} < \frac{k-1}{k}$ the SWT will have the smallest sample size with no observations outside rollout, a comparison given in the paper.

Bibliography

[1] A. J. Girling and K. Hemming. Statistical efficiency and optimal design for stepped cluster studies under linear mixed effects models. *Statistics in Medicine*, 35(13):2149, 2016.

[2] M. A. Hussey and J. P. Hughes. Design and analysis of stepped wedge cluster randomized trials. *Contemporary Clinical Trials*, 28(2):182–91, 2007.

[3] W. Woertman, E. de Hoop, M. Moerbeek, S. U. Zuidema, D. L. Gerritsen, and S. Teerenstra. Stepped wedge designs could reduce the required sample size in cluster randomized trials. *Journal of Clinical Epidemiology*, 66(7):752–8, 2013.
